# Stereotactic radiotherapy in the management of oligometastatic and recurrent head and neck cancer: a single-center experience

**DOI:** 10.1007/s00066-023-02180-9

**Published:** 2023-12-08

**Authors:** Ahmed Allam Mohamed, Miguel Goncalves, Biney Pal Singh, Mareike Tometten, Ashkan Rashad, Frank Hölzle, Stephan Hackenberg, Michael Eble

**Affiliations:** 1 Department of Radiation Oncology, RWTH Aachen University Hospital, Pauwelsstraße 30, 52074 Aachen, Germany; 2 Center for Integrated Oncology Aachen, Bonn, Cologne and Duesseldorf (CIO ABCD), Aachen, Germany; 3 Department of Oto-Rhino-Laryngology—Head and Neck Surgery, RWTH Aachen University Hospital, Aachen, Germany; 4 Department of Hematology, Oncology and Stem Cell Transplantation, RWTH Aachen University Hospital, Aachen, Germany; 5 Department of Oral and Maxillofacial Surgery, RWTH Aachen University Hospital, Aachen, Germany

**Keywords:** Neoplasm metastasis, Metastasis directed therapy, Oligometastasis, SABR

## Abstract

**Introduction:**

Oligometastatic disease (OMD) is a metastatic stage that could benefit maximally from local therapies. Patients in this state have a better prognosis relative to those with disseminated metastases. Stereotactic radiotherapy provides a non-invasive ablative tool for primary malignant tumors and metastases.

**Materials and methods:**

We searched our register for patients with oligometastatic or recurrent head and neck cancer (OMD/R-HNC) who received stereotactic radiotherapy to manage their OMD/R. We evaluated the survival outcomes and prognostic factors that affected the survival of those patients.

**Results:**

In all, 31 patients with 48 lesions met the inclusion criteria for the analysis. The lesions comprised various metastatic sites, with the majority being pulmonary (37 lesions). Squamous cell cancer was the most common histology (26 patients). The median overall survival (mOS) was 33 months, with a progression-free survival (PFS) of 9.6 months. Eight patients received subsequent stereotactic radiotherapy after disease progression. The local control (LC) rates were 91.3, 87.7, and 83% at 6, 12, and 36 months. Patients with the de novo OMD who received stereotactic radiotherapy as their initial treatment had a median systemic treatment-free survival of 23.9 months. In univariate analysis, a trend for better OS was observed in patients with p16-positive squamous cell tumors; patients who progressed within 150 days after diagnosis had a significantly lower OS. De novo OMD showed significantly better PFS compared to induced OMD. Multivariate analyses identified p16-positive squamous cell cancer, metachronous OMD and a longer time to progression as positive predictors of OS, while de novo OMD was the only positive predictor for PFS. Treatment-related toxicities were generally mild, with two cases of grade 3 dysphagia reported.

**Conclusion:**

Stereotactic radiotherapy demonstrated favorable outcomes in patients with OMD/R-HNC with limited toxicities. Further studies are warranted to validate these findings and optimize treatment strategies for this patient population.

## Introduction

Historically, recurrent or metastatic head and neck cancer (r/m HNC) was associated with a dismal prognosis and an overall survival that did not exceed a few months using standard chemotherapies [1]. A significant improvement in the survival of these patients was achieved by the addition of cetuximab, a recombinant chimeric monoclonal antibody that blocks the epidermal growth factor receptor (EGFR), to a platin-based chemotherapy combination [2]. Lately, pembrolizumab, targeting the programmed cell death protein “PD-1” receptor, as a single agent or combined with chemotherapeutics, improved the overall survival (OS) to 13 months for patients with m/r HNC in the first-line setting [3]. According to the interim analysis, pembrolizumab alone improved OS in patients with a combined positive score (CPS) ≥ 20 and ≥ 1 compared to cetuximab chemotherapy to 14.9 and 12.3 months vs. 10.8 and 10.4 months, respectively [4].

The term “oligometastatic disease” (OMD) was introduced in 1995 and defined as a disease state with limited metastases in few organs. This state exists between the non-metastatic stage and the extensive metastatic stage, and may still benefit from local curative therapies [5, 6]. The OMD is a dynamic state that can switch between a newly diagnosed OMD (de novo), recurrent OMD after local therapy (repeat OMD), or OMD after the utilization of systemic treatment (induced-OMD) [7].

One of the established local curative therapies is stereotactic ablative radiotherapy. This method entails a precise image-guided application of a high radiation dose in one or a few fractions, resulting in a highly tumoricidal effect [8, 9]. Stereotactic radiotherapy can be applied as a local ablative tool for primary tumors such as non-small cell lung cancer [10] and hepatocellular carcinoma [11]. Also, different prospective studies showed improved survival outcomes by treating OMD with stereotactic radiotherapy [12, 13]. Besides the efficacy of stereotactic radiotherapy as a metastasis-directed therapy, it may defer the systemic treatment for selected patients, reducing the potential side effects resulting from these therapies [14, 15].

Despite the scarcity of evidence, local therapies, including stereotactic radiotherapy, have been recommended to initially treat the oligometastatic or recurrent disease of head and neck cancer (OMD/R-HNC) [16].

Generally, novel combinations of targeted therapies and immunotherapy with radiation in head and neck cancer are being evaluated [17, 18], and specifically for OMD/R-HNC, the combination of pembrolizumab with SBRT is promising [19] and is currently being evaluated prospectively in the phase III ECOG-ACRIN 3211 study.

In the current retrospective study, we aimed to report on the single institution experience of stereotactic ablative radiotherapy in OMD/R-HNC, emphasizing local control outcomes as well as the overall and progression-free survivals. Furthermore, we also investigated the prognostic factors that influence survival, to enable us to define suitable candidates for local therapies in the future.

## Materials and methods

Patients with OMD/R-HNC, defined as having no more than five metastases or locoregional recurrence, were discussed in a multidisciplinary tumor board (MDT), including medical and radiation oncologists as well as head and neck surgeons. Local therapy approaches for these patients were always preferred, including radiotherapy or surgical resection if possible.

We searched our local register to identify patients with OMD/R-HNC who underwent stereotactic cranial or extracranial radiotherapy as part of their treatment between 2009 and 2022.

The inclusion criteria for the analysis were (1) synchronous de novo oligometastatic patients (defined as patients whose diagnosis as OMD was made within 4 months of the initial diagnosis), (2) metachronous de novo oligometastatic patients (defined as patients with OMD who were diagnosed after at least 4 months from the initial diagnosis), (3) locoregional recurrence, and (4) polymetastatic patients who received chemotherapy and converted to oligometastatic (induced OMD).

Exclusion criteria were (1)intentionally untreated primary tumor (as it would be a nonradical concept for treatment), (2) palliative radiotherapy for symptom relief, and (3) lacking histological confirmation, at least for the primary treatment.

### Stereotactic radiotherapy

For synchronous de novo oligometastatic patients, the metastatic site would be treated after treating the primary disease. Patients with extracranial metastases were treated with stereotactic body radiotherapy. Briefly, each patient received computerized tomography scan for planning purposes (P-CT) on a 16-slice CT scanner (Brilliance CT Big Bore Oncology, Philips Medical Systems, Inc., Cleveland, OH, USA) with vacuum cushions in breath-hold for thoracic or upper abdomen targets whenever possible. In cases where patients could not hold their breath, they underwent 4D P‑CT to delineate the moving target lesions with an additional abdominal compression being applied as passive motion management.

Patients with cranial metastases received stereotactic radiosurgery (SRS) or fractionated radiotherapy (SFR). Briefly, patients would receive histological confirmation, either by means of open surgery or stereotactic biopsy, followed by a P-CT for simulation in a stereotactic frame.

The radiation dose for SBRT/SRS/SFRT was prescribed to the isodose line (90–67%) to achieve dose-inhomogeneity in the center of the tumor [20]. The typical number of fractions would be 1–12, depending on the site, tumor size, and motion management. Lesions were counted separately if they had separate planning target volumes (PTV).

Thirty-one patients were identified in the local register with oligometastatic or recurrent head and neck tumors who received stereotactic radiotherapy throughout their treatment and met the inclusion criteria. The median prescribed dose was 45 Gy (range 60–24 Gy) with a median equivalent dose of 2 Gy per fraction (EQD2 _α/β 10_) = 88 Gy (range 93.8–31.2 Gy) and median three fractions (range 1–12 fractions).

The treatment-related toxicities were collected from the documentation of the treating physician, and their severity was graded according to Common Terminology Criteria for Adverse Events (CTCAE) version 5.

The local ethics committee approved the current retrospective study (RWTH Aachen University, Faculty of Medicine, Ethics Committee “EK-22-440”). As an individual informed consent was obtained from each patient before the treatment, a separate consent for the study was not necessary.

### Statistical analysis

The statistical analysis and graphics were executed using the R software version. The OS time was defined as the interval from the diagnosis of OMD to the time of death or censoring. Progression-free survival (PFS) was defined as the interval from the end of the radiation treatment until any site disease progression or censoring. Local control (LC) was defined as the interval from the end of the radiation treatment to the radiological progression of the irradiated lesion or censoring. Systemic therapy-free time (STFS) was defined as the interval from the end of radiation treatment to the introduction of systemic therapy by disease progression. The log-rank test was used for univariate analysis, and the Cox regression test was used for multivariate analysis. Time-dependent receiver operating characteristic curve (ROC) analysis was applied to calculate the cut-point with the most robust statistical significance.

## Results

### Patients’ characteristics

Patients’ characteristics are provided in Table 1. In total, 48 lesions were treated, including 37 pulmonary metastases, 5 mediastinal lymph node metastases, 2 spine metastases, 1 regional recurrence, 1 brain metastasis, 1 adrenal gland metastasis, and 1 renal metastasis. Twenty-six patients had squamous cell cancer histology with 41 lesions. Five patients with a nonsquamous cell cancer histology had 7 lesions: 2 patients had adenoid cystic carcinoma, 2 patients had adenocarcinoma, and 1 patient had mucoepidermoid carcinoma. Of the 26 patients with a squamous cell cancer histology, 16 patients were p16-negative, 7 patients were p16-positive, and the p16-status for 3 patients was unknown.

**Table 1:** Baseline characteristics

Characteristics	
*Number of patients*	31
*Number of lesions*	48
*Median age (range), years*	69 (45–90)
*Gender*	
Male	19
Female	12
*Primary site*	
Oropharynx	9
Hypopharynx/Larynx	7
Oral cavity	6
Salivary glands	6
Nose	1
CUP	2
*Histology*	
Squamous cell cancer	26
P16-negative	16
P16-positive	7
P16 unknown	3
Nonsquamous cell cancer	5
*OMD*	
Synchronous	8
Metachronous	23 (include 1 oligorecurrent)
*Treatment of primary*	
Primary chemoradiotherapy	11
Surgery + adjuvant radiotherapy	11
Surgery + adjuvant chemoradiotherapy	4
Surgery	3
Radiotherapy	2
*Treated metastatic lesions (total)*	48
Pulmonary	37
Lymph node	5
Spine	2
Adrenal gland	1
Renal	1
Brain	1
Regional recurrence	1
*Median dose as EQD2 *_*α/β 10*_* (range), Gy*	88 (31.2–93.8)
*Median number of fractions (range)*	3 (1–12)
*Prior chemotherapy for the metastatic disease*	
Yes (induced OMD)	3
No (de novo)	28
*Systemic therapy during metastatic stage*	
Chemotherapeutics (include cetuximab)	12
Immunotherapy (anti-PD-1)	6
None	16

*OMD* oligometastatic disease, *Gy* Gray, *CUP* cancer of unknown primary, *EQD2* median equivalent dose in 2 Gy per fraction

Regarding the type of OMD, 28 patients had de novo OMD; this included 8 patients with synchronous OMD and 23 patients with metachronous OMD, including 1 patient with regional recurrence. Three patients previously had metachronous polymetastatic disease, received systemic therapy, converted to OMD (induced OMD), and subsequently received stereotactic radiotherapy to the oligometastatic lesions.

### Survival outcomes

**Fig. 1:**
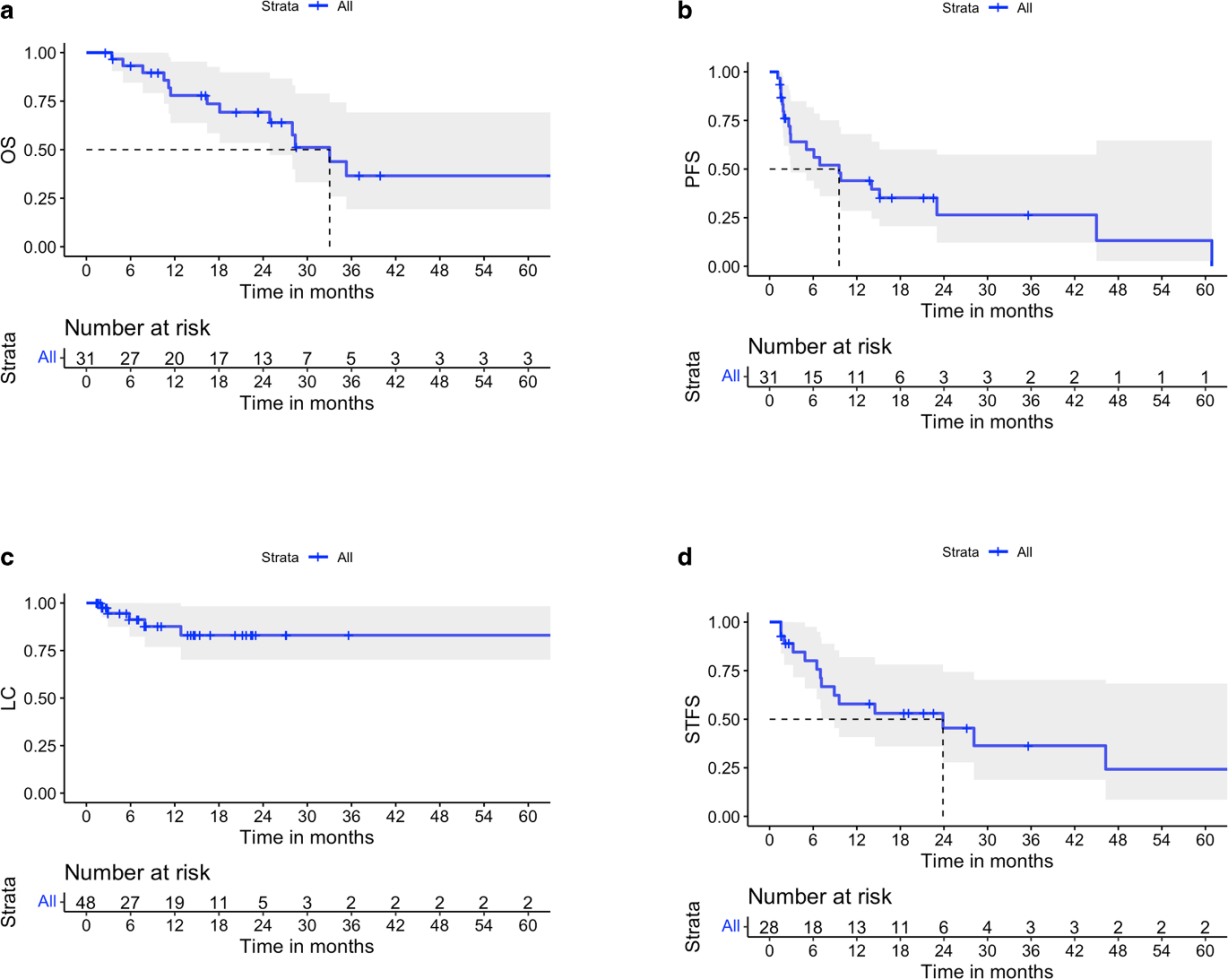
Kaplan–Meier curves presenting **a** overall survival (OS) of patients with oligometastatic disease (OMD) who received stereotactic radiotherapy, **b** progression-free survival (PFS) of the entire cohort, **c** local control (LC) rate of treated lesions, **d** time to introduction of systemic treatment, systemic treatment-free survival (STFS), for de novo OMD patients. *grey curves* present the 95% confidence interval

After 28.5 months of median follow-up, 12 of 31 patients had passed away, and the median overall survival time was 33 months. The OS rate at 1 year was 77.9%, and at 3 and 5 years it was 36.5% (Fig. 1a). The median OS for de novo OMD and induced OMD were 33 and 24.9 months, respectively.

Fifteen patients experienced a systemic progression after stereotactic radiotherapy with a median PFS of 9.6 months; subsequent SBRT after disease progression was applied in 8 patients (Fig. 1b).

Out of 48 lesions treated with stereotactic radiotherapy, 5 patients experienced local failure. The local control rates at 6, 12, and 36 months were 91.3, 87.7, and 83%, respectively (Fig. 1c).

For patients who had not received any systemic treatment after being diagnosed with the metastatic disease and who had received stereotactic radiotherapy first (de novo OMD), the median STFS was 23.9 months (Fig. 1d).

### Prognostic factors (univariate and multivariate analyses)

**Fig. 2:**
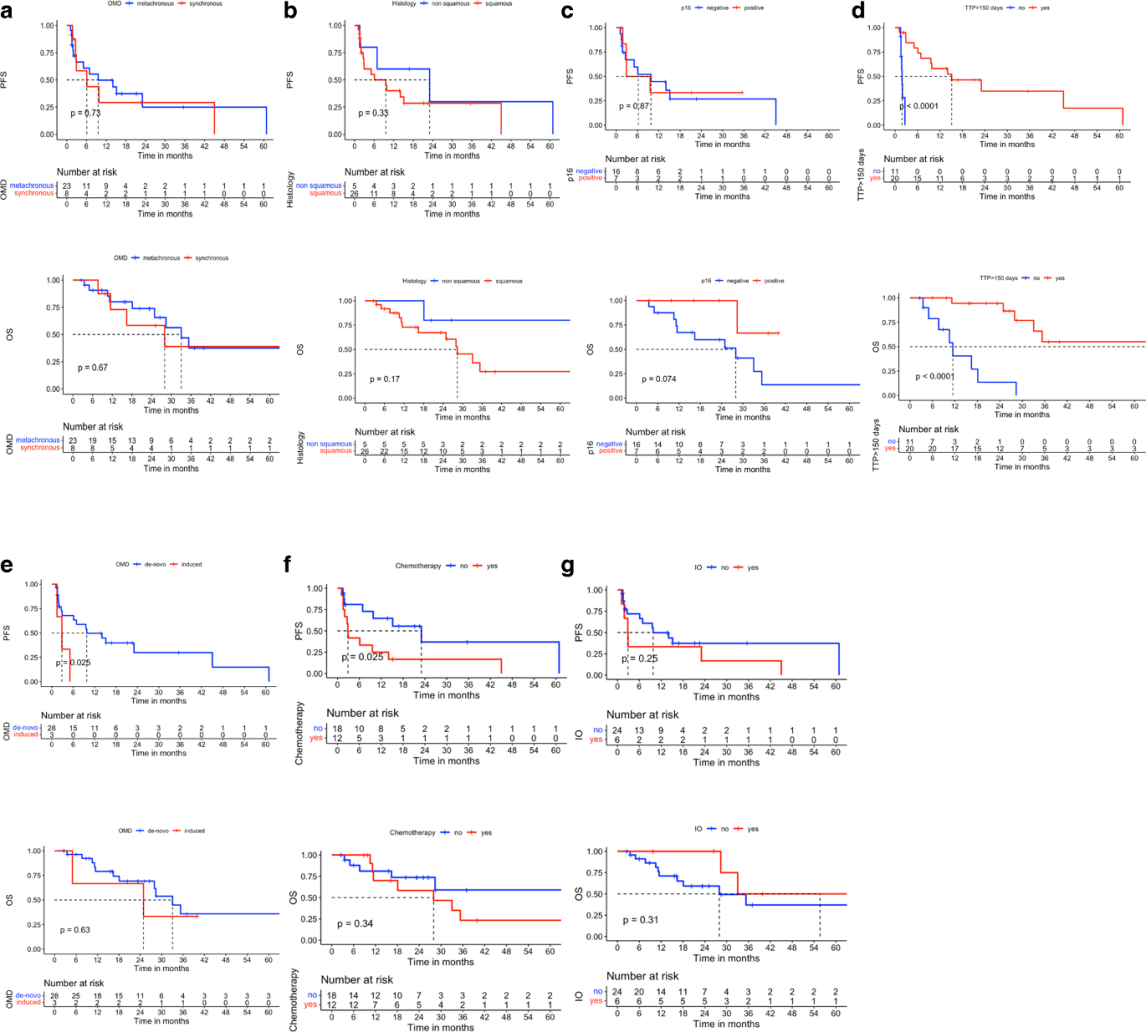
Univariate analysis using Kaplan–Meier curves with log-rank test for progression-free survival (PFS) and overall survival (OS) based on **a** oligometastatic disease (OMD) type (metachronous vs. synchronous), **b** histology (squamous cell cancer vs. nonsquamous cell cancer), **c** p16 (positive vs. negative), **d** time to progression (TTP) > 150 days (yes vs. no), **e** de novo OMD vs. induced OMD, **f** those who received chemotherapy throughout metastatic disease vs those who did not receive chemotherapy, **g** those who received immunotherapy (IO)

A univariate analysis was conducted using the log-rank test to investigate the prognostic factors influencing the survival outcomes for OMD/R-HNC patients. Synchronous and metachronous OMD showed no significant difference regarding OS or PFS (*p*-value: 0.67 and 0.73, respectively; Fig. 2a). In addition, squamous cell cancer and nonsquamous cell cancer histologies did not influence OS and PFS (Fig. 2b). However, we observed a trend for better OS in patients with p16-positive tumors compared to p16-negative tumors, specifically among those with a squamous cell cancer histology (*p*-value 0.074; Fig. 2c). We also examined the impact of the time to progression (TTP) of OMD from the date of diagnosis using the time-dependent ROC curve analysis between the TTP and OS. We found that a cut-point of TTP < 150 days showed the most robust significant survival difference. Patients who progressed within 150 days after diagnosis (TTP < 150 days) had, besides a lower PFS, also a significantly lower OS (*p*-value < 0.001; Fig. 2d). In addition, a de novo OMD showed a significantly better PFS than the induced OMD (*p*-value = 0.025). However, neither had a meaningful statistical difference in OS (*p*-value = 0.63; Fig. 2e). Understandably, patients who received chemotherapeutics (including cetuximab) throughout their metastatic disease had a significantly lower PFS (*p*-value = 0.025). However, the OS difference with those not receiving the chemotherapeutics was statistically nonsignificant (*p*-value = 0.34; Fig. 2f). Furthermore, patients who received immunotherapy (anti-PD-1) throughout the treatment of their metastatic disease did not show a significant improvement for either PFS or OS (*p*-value = 0.25 and 0.31, respectively; Fig. 2g).

There was no statistical difference in local control between treated pulmonary and non-pulmonary lesions (*p*-value = 0.93), nor was there a statistically significant difference based on the histology of the lesion (squamous vs. nonsquamous; *p*-value = 0.48). ROC analysis showed the greatest impact on local control was at a dose cut-point of EQD2 at 87.25 Gy. However, there was no statistically significant difference in local control between doses lower or higher than 87.25 Gy (*p*-value = 0.17).

**Fig. 3:**
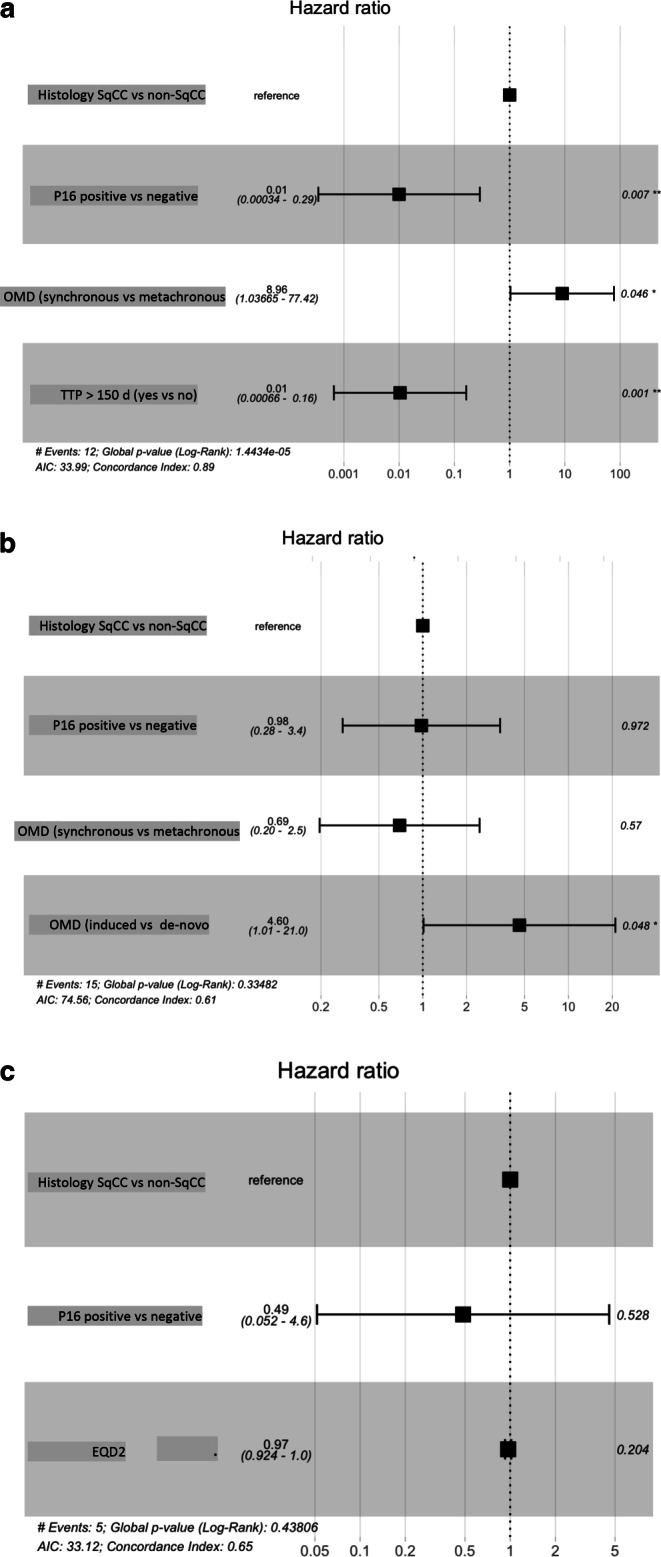
Cox regression models for **a** overall survival (OS), **b** progression-free survival (PFS), and **c** local control (LC). *SqCC* squamous cell cancer, *TTP* time to progression, *OMD* oligometastatic disease, *EQD2* equivalent dose in 2 Gy per fraction. *p-value < 0.05, **p-value < 0.001

Using Cox regression for the multivariate analysis, p16-positive squamous cell cancer, metachronous OMD, and TTP are positive predictors for OS (Fig. 3a). Furthermore, de novo OMD was the only positive predictor in the Cox regression model for PFS (Fig. 3b). Lastly, radiation dose (EQD2), histology, and p16-status were not predictors for local control (Fig. 3c).

### Treatment-related severe toxicities

Two patients (6.5%) suffered from grade 3 dysphagia: 1 patient after SBRT of regional recurrence and 1 patient after repeated SBRT of mediastinal metastatic lymph nodes. No grade 5 toxicity was documented from the treatment.

## Discussion

In their groundbreaking editorial, Hellman and Weichselbaum [5] described a disease state existing between the non-metastatic and the disseminated metastatic stages and gave it the term “oligometastases”. They promulgated the benefit of local therapies for this stage in improving survival outcomes. Consequently, several studies addressed stereotactic ablative radiotherapy in OMD, confirming the survival advantage of the approach [12, 13, 21]. In addition to its effectiveness, stereotactic radiotherapy is also advantageous because it is non-invasive.

The current analysis offers insight into the treatment outcomes and factors that predict the prognosis for patients with oligometastatic or recurrent head and neck tumors who underwent stereotactic radiotherapy. According to our study, this treatment approach could improve survival rates among the oligometastatic patient group. Only a handful of studies have addressed the topic of stereotactic radiotherapy in OMD-HNC (Table 2; [22–26]).

**Table 2:** Studies that examined the role of stereotactic radiotherapy in oligometastatic or recurrent head and neck tumors

Author	Type of study	Number of patients	Site of metastases	Survival outcomes
Bonomo et al. [22]	Retrospective	27	28 pulmonary (100%)	ORR at 3 months after SBRT: 75%
				Median PFS: 16 months
				Median OS of 47 months
Bates et al. [23]	Retrospective	27	Pulmonary: 16 (59.3%) Other sites:11 (40.7%)	Median OS: 1.9 years
				Median PFS: 0.5 years
				The 1‑ and 2‑year LC rates: 75% and 57%
Weissmann et al. [24]	Retrospective	Total 40 (90% radiotherapy)	Pulmonary 58%	Median OS 23.0 months
				Median PFS for patients with radiation 9.9 months
				1 and 2‑year LC was 90%
Pasalic et al. [25]	Retrospective	82	107 pulmonary (100%)	Median follow-up 20 months, LC, and OS rates were 94%, and 62%
Franzeese et al. [26]	Retrospective	48	71 lesions: lung 59.1%, bone 15.5%, lymph node 14.1% liver 7.1% and adrenal gland 4.2%	LC at 1 and 2 years were 83.1% and 70.2%
				PFS at 1 and 2 years were 42.2% and 20.0%
				OS rates at 1 and 2 years were 81.0% & 67.1%

*ORR* overall response rate, *SBRT* stereotactic body radiotherapy, *PFS* progression-free survival, *OS* overall survival

This study involved 31 patients with a median age of 69 years. The lesions treated were in various sites, but most were pulmonary metastases. Most patients had squamous cell cancer (83.8%). However, other nonsquamous cell cancers were also included, such as adenoid cystic, adeno-, and mucoepidermoid carcinoma. The inclusion of different types of tumor histologies may broaden the applicability of the results generally in OMD/R-HNC, though patient numbers are low.

Based on the results, stereotactic radiotherapy successfully controlled most treated lesions. Out of 48 lesions, only 5 patients experienced local failure, resulting in LC rates of 91.3, 87.7, and 83% at 6, 12, and 36 months, respectively. These findings support previous studies that have shown the effectiveness of stereotactic radiotherapy in achieving high rates of local control [24–26].

Regarding the survival outcomes, the median OS in the study cohort was 33 months (33 months for de novo OMD and 24.9 months for induced OMD), with an OS rate of 77.9% at 1 year and 36.5% at 3 and 5 years. The findings validate the survival benefits of stereotactic radiotherapy in the same patient populations as in previous studies (Table 2), suggesting that almost one-third of these patients may experience an extended survival of more than 3 years.

Also, 15 patients experienced systemic progression after undergoing stereotactic radiotherapy, with the median PFS being 9.6 months. It is worth noting that 8 of those patients received stereotactic radiotherapy after their disease progressed, suggesting that local therapies, including stereotactic radiotherapy, could still be applied in cases of further progression after the initial local treatment of OMD.

Based on the findings presented in Keynote 048, which showed that the administration of pembrolizumab led to a 13-month OS and 2.3-month PFS when used as the first-line systemic treatment for r/m HNC [3, 4], it appears that stereotactic radiotherapy could potentially improve the OS of patients with oligometastatic or recurrent head and neck tumors. However, it is clear that most of patients in Keynote 048 had advanced or disseminated r/m HNC with a higher tumor burden compared to OMD/R-HNC.

To determine factors that affect survival outcomes univariate analyses were conducted. The results revealed that the type of oligometastatic disease (synchronous or metachronous) and histology (squamous vs. nonsquamous) had a prognostic effect neither for the OS nor for the PFS. However, a trend towards a better OS was observed in patients with p16-positive squamous cell tumors. This finding is consistent with previous studies that have reported a favorable prognosis in p16-positive head and neck cancer patients [27].

In addition, the TTP of OMD-HNC was found to be a significant prognostic factor. Patients who experienced disease progression within 150 days after OMD diagnosis had a significantly lower OS. Furthermore, the de novo OMD showed significantly better PFS than induced OMD, indicating that induced OMD is a disease state mostly with widespread occult metastases and, for this scenario, providing local therapies such as stereotactic radiotherapy under the umbrella of systemic treatment would improve the outcomes. Mainly due to the limited number of patients in the study, we could not find a statistical advantage for the administration of chemotherapeutics or immunotherapy during the OMD/R stage for OS.

Regarding the multivariate analysis, we found that a better OS has been associated with p16-positive tumors, metachronous OMD, and TTP > 150 days. Furthermore, de novo OMD was the only factor associated with a better PFS. Finally, a factor that may influence the Cox regression model for LC could not be determined.

## Limitations of the study

This study has two shortcomings that should be openly addressed. First, the sample size was relatively small, so the survival findings may not necessarily apply to a broader population. Second, the study design was retrospective, which could lead to the introduction of selection bias and confounding factors. More extensive prospective studies are necessary to confirm these findings and explore the prognostic factors for survival outcomes in patients with OMD/R-HNC local therapies such as stereotactic radiotherapy.

## Conclusion

The findings of this study suggest that stereotactic radiotherapy is an effective treatment approach for OMD/R-HNC. It demonstrates favorable survival outcomes and high rates of local control. Prognostic factors such as p16-status, type of OMD, and time to progression have been identified as potential predictors of survival outcomes. These findings provide valuable insights for clinical decision-making and may help optimize treatment strategies for the OMD/R-HNC patient population. Further research with larger prospective studies is needed to validate these findings and explore additional factors that may influence treatment outcomes.
